# Mural Unicystic Ameloblastoma in a 62‐Year‐Old Patient Mimicking Conventional Ameloblastoma: A Case Report

**DOI:** 10.1155/crid/2912131

**Published:** 2026-07-09

**Authors:** Faisal Mehsen Alali, Maya Al-jokhadar, Mohammad Al-Qudsi, Ali Alrafedah, Mohammed Alshammari, Yousef Alkhaibari, Bassel Tarakji

**Affiliations:** ^1^ Department of Oral Maxillofacial Surgery and Diagnostic Sciences, College of Dentistry, Prince Sattam Bin Abdulaziz University, Al kharj, Riyadh Province, Saudi Arabia, psau.edu.sa; ^2^ Department of Oral Histology and Pathology, College of Dentistry, Arab International University, Daraa, Daraa Governorate, Syria, aiu.edu.sy; ^3^ Department of Oral and Maxillofacial Surgery, Ibn Al-Nafees Hospital, Damascus, Damascus Governorate, Syria

**Keywords:** conventional ameloblastoma, intraluminal plexiform proliferation, multilocular radiolucency, mural unicystic ameloblastoma, unicystic ameloblastoma

## Abstract

Unicystic ameloblastoma is a cystic variant of ameloblastoma that may clinically and radiographically mimic odontogenic cysts and, in some cases, conventional ameloblastoma. The mural subtype is characterized by ameloblastomatous epithelial infiltration of the fibrous cyst wall and may demonstrate more aggressive biological behavior than other unicystic variants. This report describes a mural unicystic ameloblastoma in a 62‐year‐old male presenting with a long‐standing painless swelling of the left mandible. Cone‐beam computed tomography demonstrated a well‐defined radiolucent lesion measuring approximately 47 × 25 mm with marked buccolingual expansion, internal septations producing a multiloculated appearance, and focal buccal cortical perforation. Incisional biopsy established the diagnosis of ameloblastoma without definitive subclassification. Segmental mandibular resection was subsequently performed because of the lesion′s radiographic extent and cortical perforation. Histopathological examination of the resected specimen demonstrated a true cystic lesion lined by ameloblastomatous odontogenic epithelium with extensive intraluminal plexiform epithelial proliferation and multiple ameloblastomatous epithelial islands infiltrating the fibrous cyst wall, confirming mural unicystic ameloblastoma. Despite these findings, preservation of the underlying cystic architecture supported the diagnosis of mural unicystic ameloblastoma rather than predominantly solid ameloblastoma with secondary cystic degeneration. This case highlights the diagnostic challenges of unicystic ameloblastoma in lesions demonstrating multiloculated radiographic appearance, cortical perforation, and extensive intraluminal and mural proliferation, and emphasizes the importance of adequate histopathological sampling and clinicopathological correlation for accurate subclassification and surgical management.

## 1. Introduction

Ameloblastoma is an odontogenic epithelial neoplasm characterized by locally invasive growth, progressive bone destruction, and a recognized tendency for recurrence [1, 2]. The current World Health Organization classification recognizes several clinicopathological variants of ameloblastoma, including solid/multicystic, unicystic, and peripheral types [3]. Unicystic ameloblastoma is recognized as a distinct clinicopathological variant that generally demonstrates less aggressive behavior than conventional ameloblastoma; however, delayed diagnosis may permit progressive enlargement and the development of locally destructive lesions with increased diagnostic and surgical complexity [1, 4]. Radiographically, unicystic ameloblastoma most commonly presents as a well‐defined unilocular radiolucency that may mimic odontogenic cystic lesions, often creating diagnostic uncertainty [5, 6]. Preoperative incisional biopsy may underestimate the full architectural complexity of unicystic ameloblastoma, particularly in lesions with focal mural invasion or heterogeneous epithelial proliferation, thereby limiting accurate subclassification before definitive surgical treatment [7]. Histopathologically, unicystic ameloblastoma is subclassified into luminal, intraluminal, and mural variants according to the extent and pattern of ameloblastomatous epithelial proliferation [3]. Among these variants, the mural subtype is clinically significant because infiltration of the fibrous cyst wall is associated with more aggressive biological behavior and an increased risk of recurrence [2, 8]. Although unicystic ameloblastoma may occur over a broad age range, most cases are reported in younger patients, whereas presentation in elderly individuals remains relatively uncommon [3, 7, 9]. In the present case, advanced patient age, multiloculated radiographic appearance, cortical perforation, prominent intraluminal plexiform proliferation, and mural tumor infiltration coexisted within a single lesion, creating an unusual clinicopathological constellation that closely mimicked conventional ameloblastoma and complicated both diagnosis and therapeutic decision‐making. This case highlights the diagnostic challenges that may arise when multiple atypical clinicoradiographic and histopathological features coexist within a unicystic ameloblastoma and underscores the importance of extensive histopathological sampling, careful radiographic–histopathological correlation, and accurate subclassification for appropriate surgical management. This case report was prepared in accordance with the CARE reporting guidelines.

## 2. Case Presentation

A 62‐year‐old male was referred to the oral and maxillofacial surgery service at a tertiary referral hospital after initial evaluation at a peripheral healthcare center. The patient reported a long‐standing history of gradually progressive mandibular enlargement over several years. The lesion was largely asymptomatic, with no associated pain, sensory disturbance, or signs of acute inflammation. The medical history was noncontributory. Clinical examination revealed facial asymmetry secondary to significant bony expansion. Intraoral evaluation demonstrated cortical expansion without mucosal ulceration or surface alterations. The overlying mucosa appeared clinically unremarkable. Radiographic evaluation was performed using cone‐beam computed tomography (CBCT), which demonstrated a well‐defined radiolucent lesion involving the left posterior mandible. The lesion measured approximately 47 × 25 mm in its greatest radiographic dimensions and appeared predominantly cystic, exhibiting marked buccolingual expansion and internal septations imparting a multiloculated appearance. The lesion also demonstrated perforation of the buccal cortical plate, with focal discontinuity of the cortical outline and extension toward adjacent soft tissues. Radiographic characteristics of the lesion are presented in Figure 1A,B. An incisional biopsy had previously been performed at the referring peripheral center and established the diagnosis of ameloblastoma without subtype specification. The limited biopsy material and the heterogeneous architecture of the lesion likely precluded definitive histopathological subclassification, as accurate subclassification of unicystic ameloblastoma may require examination of the entire lesion [4]. Given the considerable size and extent of the lesion, the multiloculated radiographic appearance, cortical perforation, and extension toward adjacent soft tissues, surgical management under general anesthesia was undertaken. A segmental/hemimandibular resection extending from the left mandibular angle to the midline was carried out. Mandibular continuity was restored using a titanium reconstruction plate without immediate prosthetic rehabilitation or temporary prosthetic replacement. Gross examination revealed a mandibular resection specimen measuring 130 × 32.3 × 38 mm. Gross features of the resected specimen are illustrated in Figure 2. On sectioning, the lesion appeared predominantly cystic and contained hemorrhagic material. Gross examination also confirmed perforation of the buccal cortical plate. Following decalcification, the specimen was serially sectioned into three large representative tissue segments. Multiple histopathological sections were subsequently obtained from different areas of the lesion, including multiple central regions as well as the anterior and posterior surgical margins, allowing adequate evaluation of the cystic architecture, intraluminal proliferation, mural infiltration, and surgical margin status. Despite extensive sampling from multiple representative regions of the lesion, no separate solid ameloblastomatous tumor component independent from the cystic architecture was identified. Microscopic examination demonstrated a large cystic cavity lined by odontogenic epithelium with extensive intraluminal plexiform epithelial proliferations projecting into the cystic lumen (Figure 3A–C). These intraluminal proliferations formed multiple plexiform epithelial nodules and were consistent with the recognized intraluminal subtype, in which plexiform proliferation commonly predominates [10, 11]. Examination of other areas of the lesion demonstrated preservation of the true cystic architecture, with a recognizable luminal lining composed of palisaded basal ameloblast‐like cells with reverse polarization and overlying stellate reticulum–like epithelium (Figure 4A–C). Multiple ameloblastomatous epithelial islands and cords infiltrating the fibrous cyst wall were also identified, consistent with mural unicystic ameloblastoma (Figures 4C). Despite the extensive intraluminal and mural proliferation, the persistence of recognizable luminal cystic lining in multiple areas supported the diagnosis of mural unicystic ameloblastoma rather than predominantly solid ameloblastoma with secondary cystic degeneration. Sections obtained from the surgical margins revealed normal bone and soft tissue without evidence of tumor infiltration, confirming clear surgical margins of approximately 8 mm. Postoperative healing was satisfactory. Speech and swallowing functions remained preserved because the tongue was not involved surgically. Occlusion was maintained only on the contralateral nonresected side because no immediate prosthetic rehabilitation was performed. The aesthetic outcome was considered acceptable. Long‐term postoperative follow‐up was limited because the patient resided in a geographically remote and medically underserved area far from the referral center where treatment was performed, with significant transportation and communication limitations. Several attempts were made to obtain follow‐up information by telephone; however, successful contact was achieved on only one occasion, during which the patient reported no major postoperative complaints. The chronological sequence of the patient′s clinical course is summarized in Table 1.

**Figure 1:**
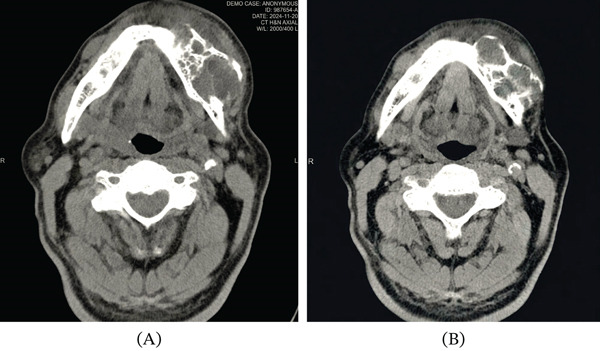
(A,B)Axial cone‐beam computed tomography (CBCT) images (bone window) demonstrating a multiloculated radiolucent lesion involving the left mandible. The lesion shows marked buccolingual expansion with thinning and focal perforation of the buccal cortical plate. Multiple internal compartments are evident, producing a multilocular appearance. The lesion measured approximately 47 × 25 mm in its greatest radiographic dimensions.

**Figure 2:**
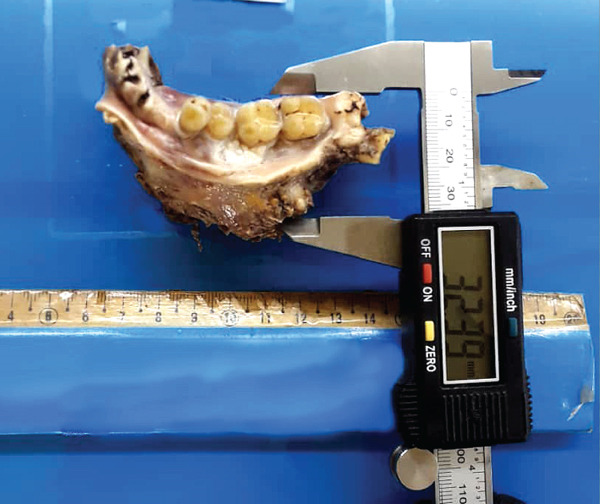
Gross photograph of the resected mandibular segment following en bloc surgical resection. The specimen measured approximately 130 × 32.3 × 38 mm. A digital caliper demonstrates one dimension (32.39 mm), corresponding to the measurement documented in the histopathological report. The specimen includes a segment of mandible with associated teeth.

**Figure 3:**
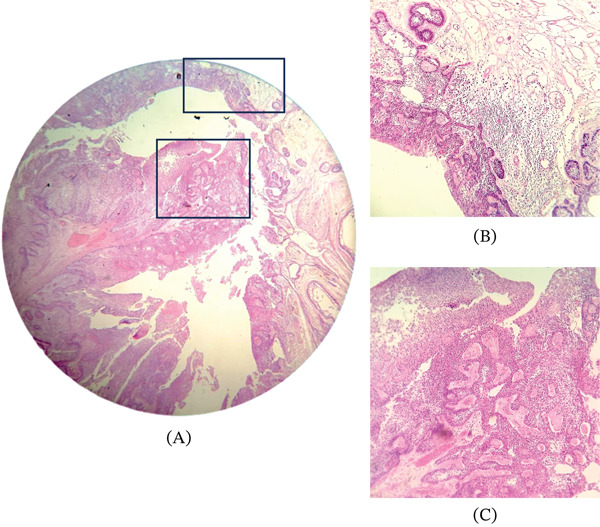
Histopathological features of the first representative microscopic field demonstrating the overall cystic architecture and the relationship between the intraluminal and mural components of mural unicystic ameloblastoma. (A) Low‐power view (×4) showing a large cystic cavity lined by odontogenic epithelium with extensive intraluminal plexiform epithelial proliferation projecting into the cystic lumen and associated mural ameloblastomatous epithelial proliferations within the fibrous cyst wall. The boxed areas indicate the regions shown at higher magnification in Panels B and C. (B) Low‐power view (×10) of the upper boxed region shown in Panel A, demonstrating proliferative ameloblastic cystic lining associated with extensive intraluminal plexiform epithelial proliferation and adjacent mural ameloblastomatous epithelial islands within the fibrous cyst wall, illustrating the coexistence of the luminal, intraluminal, and mural components in the same representative field. (C) Low‐power view (×10) of the central boxed region shown in Panel A, highlighting a prominent plexiform intraluminal epithelial proliferation projecting into the cystic lumen together with adjacent mural ameloblastomatous epithelial proliferations within the fibrous cyst wall, illustrating the transition between the intraluminal and mural components.

**Figure 4:**
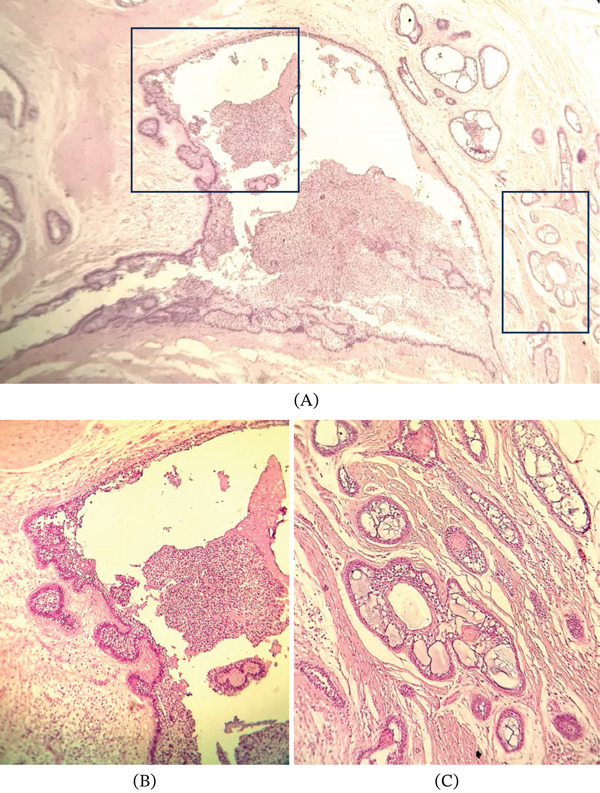
Histopathological features of a second representative microscopic field demonstrating the classical ameloblastomatous cyst lining and mural epithelial proliferation in mural unicystic ameloblastoma. (A) Low‐power view (×10) showing a portion of the main cystic cavity lined by odontogenic epithelium with a palisaded basal cell layer and overlying stellate reticulum‐like epithelium, demonstrating preservation of the true cystic architecture. Multiple mural ameloblastomatous epithelial islands are also present within the fibrous cyst wall. The left black box indicates the region shown at higher magnification in Panel B, whereas the right black box indicates the region shown in Panel C. (B) Medium‐power view (×20) representing a higher magnification of the region indicated by the left black box in Panel A, demonstrating a preserved ameloblastic cystic lining composed of basal ameloblast‐like cells with reverse nuclear polarity and overlying stellate reticulum‐like cells. (C) Medium‐power view (×20) representing a higher magnification of the region indicated by the right black box in Panel A, demonstrating extensive ameloblastomatous epithelial islands and cords within the fibrous cyst wall, consistent with mural unicystic ameloblastoma.

**Table 1:** Clinical timeline of the diagnostic, therapeutic, and follow‐up course.

Time point	Clinical event
Several years before presentation	Gradual painless enlargement of the left mandible.
Initial evaluation	Assessment at a peripheral healthcare center.
Preoperative diagnosis	Incisional biopsy established the diagnosis of ameloblastoma without subtype specification.
Radiographic assessment	CBCT demonstrated a predominantly cystic multiloculated radiolucent lesion with cortical perforation.
Surgical management	Segmental/hemimandibular resection with placement of a titanium reconstruction plate.
Histopathological diagnosis	Predominantly cystic ameloblastomatous lesion with extensive intraluminal plexiform proliferation, mural tumor infiltration, and preservation of true cystic architecture, consistent with mural unicystic ameloblastoma.
Postoperative course	Satisfactory healing with preserved speech and swallowing function and an acceptable aesthetic outcome.
Follow‐up	Limited long‐term follow‐up; one successful telephone contact revealed no major postoperative complaints.

## 3. Discussion

This case illustrates an unusual clinicopathological presentation of mural unicystic ameloblastoma in an elderly patient, in which multilocularity, cortical perforation, extensive intraluminal plexiform proliferation, and mural tumor infiltration collectively created substantial diagnostic overlap with conventional ameloblastoma. Although unicystic ameloblastoma is generally regarded as a less aggressive variant, mural lesions demonstrate true neoplastic infiltration of the fibrous cyst wall and have been associated with more aggressive biological behavior and higher recurrence rates following conservative treatment [8, 12]. Patient age represented an additional diagnostically significant feature in the present case. Unicystic ameloblastoma predominantly affects younger individuals and is most commonly reported during the second and third decades of life, whereas presentation in elderly patients remains distinctly uncommon [7, 9, 12, 13]. In the current case, the occurrence of mural unicystic ameloblastoma in a 62‐year‐old patient, together with multilocularity and cortical perforation, further contributed to the clinicoradiographic resemblance to conventional ameloblastoma. Although the precise duration of tumor development cannot be determined retrospectively, the available clinical history does not support a lesion that had remained asymptomatic for several decades. Instead, the considerable lesion size more likely reflects progressive enlargement over several years prior to clinical presentation. The substantial size of the lesion represented another important feature contributing to the aggressive clinicoradiographic profile of the present case. Although unicystic ameloblastoma often demonstrates relatively slow enlargement, delayed clinical presentation may permit progressive expansion and the development of locally destructive lesions [1, 14]. In addition to marked buccolingual expansion, the lesion demonstrated frank cortical perforation, a less common finding in unicystic ameloblastoma that may indicate more biologically active behavior, particularly in mural lesions [8, 12]. In the present case, lesion size and cortical perforation, together with multilocularity and mural invasion, contributed substantially to the diagnostic overlap with conventional ameloblastoma and reinforced the rationale for radical surgical management rather than conservative treatment [12, 15, 16]. Radiographically, unicystic ameloblastoma most commonly presents as a well‐defined unilocular radiolucency; however, multiloculated patterns have also been described [17]. In the current case, the multiloculated radiographic appearance with internal septations increased the radiographic resemblance to conventional ameloblastoma and contributed substantially to the initial diagnostic uncertainty, reflecting the recognized radiographic overlap that may occur among ameloblastoma variants and other odontogenic lesions [9]. The multiloculated radiographic pattern observed in the present case may reflect the large predominantly cystic architecture of the lesion in association with internal septations and extensive intraluminal and mural epithelial proliferation rather than a predominantly solid tumor mass. Histopathological interpretation was further complicated by the presence of extensive intraluminal plexiform proliferation and active mural tumor infiltration, features that closely simulated conventional ameloblastoma. However, examination of the resection specimen demonstrated that the lesion retained a dominant true cystic architecture with preserved luminal lining in multiple areas (Figure 4A) in association with extensive intraluminal plexiform proliferation projecting into the main cystic cavity (Figure 3A), supporting the diagnosis of mural unicystic ameloblastoma rather than predominantly solid ameloblastoma with secondary cystic degeneration. This architectural complexity further illustrates how extensive mural and intraluminal proliferation may obscure the underlying cystic configuration of unicystic ameloblastoma and complicate accurate subclassification in limited incisional biopsy specimens. This likely explains why the initial biopsy performed at the referring peripheral center established only a general diagnosis of ameloblastoma without definitive subtype specification. Similar diagnostic limitations associated with incisional biopsy sampling in unicystic ameloblastoma have been emphasized in previous studies [5, 12, 17]. Therapeutic management of unicystic ameloblastoma should be guided by both histopathological subtype and clinicoradiographic behavior. Conservative approaches are generally considered more appropriate for luminal and intraluminal variants because these lesions are usually confined to the cystic lining and lumen, whereas mural unicystic ameloblastoma has been associated with higher recurrence rates following conservative treatment [8, 15, 18]. In the present case, mural invasion, substantial lesion size, multiloculated radiographic appearance, cortical perforation, and extension toward adjacent soft tissues collectively supported the decision to perform radical surgical resection. Because mural unicystic ameloblastoma carries a recognized risk of recurrence, long‐term postoperative surveillance remains important. In the present case, prolonged follow‐up was limited because the patient resided in a geographically remote area.

## 4. Conclusion

Mural unicystic ameloblastoma may closely simulate conventional ameloblastoma, particularly in elderly patients presenting with multilocularity, cortical perforation, extensive intraluminal plexiform proliferation, and extensive mural infiltration. This case highlights the importance of extensive histopathological sampling and careful clinicoradiographic correlation for accurate subclassification and appropriate surgical management. Long‐term follow‐up remains important because of the recognized recurrence potential of mural lesions.

## Author Contributions

Conceptualization and manuscript drafting: Faisal Mehsen Alali. Histopathological interpretation: Faisal Mehsen Alali and Maya Al‐jokhadar. Surgical management and clinical data acquisition: Mohammad Al‐Qudsi. Radiographic evaluation and manuscript review: Ali Alrafedah and Yousef Alkhaibari. Manuscript review and editing: Mohammed Alshammari and Bassel Tarakji. Supervision: Bassel Tarakji.

## Funding

No funding was received for this manuscript.

## Disclosure

All authors have read and approved the final version of the manuscript. The corresponding author had full access to all available data and takes full responsibility for the integrity and accuracy of the presented information.

## Ethics Statement

Ethical approval was not required for this study because it represents a single anonymized case report with written informed consent obtained from the patient for publication.

## Consent

Written informed consent was obtained from the patient for publication of this case report.

## Conflicts of Interest

The authors declare no conflicts of interest.

## Data Availability

The data supporting the findings of this study are available from the corresponding author upon reasonable request.
